# Learning deficits in rats overexpressing the dopamine transporter

**DOI:** 10.1038/s41598-018-32608-7

**Published:** 2018-09-21

**Authors:** Nadine Bernhardt, Maike Kristin Lieser, Elizabeth-Barroeta Hlusicka, Bettina Habelt, Franziska Wieske, Henriette Edemann-Callesen, Alexander Garthe, Christine Winter

**Affiliations:** 1 Department of Psychiatry and Psychotherapy, University Hospital Carl Gustav Carus, Technische Universität Dresden, Dresden, Germany; 2 Department of Psychiatry and Psychotherapy, Charité Universitätsmedizin Berlin, Berlin, Germany; 3 International Graduate Program Medical Neurosciences, Charité Universitätsmedizin Berlin, Berlin, Germany; 4 German Center for Neurodegenerative Diseases (DZNE) Dresden, Dresden, Germany

## Abstract

With its capacity to modulate motor control and motivational as well as cognitive functions dopamine is implicated in numerous neuropsychiatric diseases. The present study investigated whether an imbalance in dopamine homeostasis as evident in the dopamine overexpressing rat model (DAT-tg), results in learning and memory deficits associated with changes in adult hippocampal neurogenesis. Adult DAT-tg and control rats were subjected to the Morris water maze, the radial arm maze and a discrimination reversal paradigm and newly generated neurons in hippocampal circuitry were investigated post mortem. DAT-tg rats were found to exhibit a striking inability to acquire information and deploy spatial search strategies. At the same time, reduced integration of adult-born neurons in hippocampal circuitry was observed, which together with changes in striatal dopamine signalling might explain behavioural deficits.

## Introduction

Midbrain neurons located in the substantia nigra pars compacta (SNc) and ventral tegmental area (VTA)^1^ provide a ‘tonic’ baseline level and ‘phasic’ large changes of dopamine (DA) concentrations to downstream cortical and subcortical structures^2,3^. DA is released after reinforcing stimuli and novel experiences^4^ and is fundamental for cognition-related brain functions through its modulation of motivation, memory, motor output, and neuroendocrine integration.

Within the striatum, DA firing encodes errors in reward prediction, providing a learning signal to guide future behavior^5^. Striatal DA contributes to formation and expression of associations^6,7^, action selection and modulation of motivation^8,9^ together supporting learning and goal-directed behaviour.

In the hippocampus DA release occurs following novelty exposure^10^ and affects plasticity, synaptic transmission and the network activity within hippocampal circuitry^11–14^. Primarily through D1-class receptor activation, hippocampal DA release facilitates long-term potentiation^15,16^ thereby stabilizing new place maps necessary for spatial learning^17^.

Hippocampal and striatal memory systems have long been thought to operate independently. Recently, however they have been shown to act in synergism^18^. Animal studies demonstrate that hippocampal oscillatory activity increases during place learning and that hippocampal-striatal coherence appears after training, a mechanism considered necessary in switching from place learning to the usage of a proper response strategy^19^.

Further, DA is an important component of neurogenic niche signals and influences several aspects of neurogenesis including proliferation, migration and differentiation^20^ associated to hippocampal learning^21^. DA not only modulates ontogenetic neurogenesis^22^, in the adult brain DA fibres directly target subventricular zone (SVZ) and subgranular zone (SGZ) neuronal precursors^23,24^ expressing DA receptors^24–26^. In this line ablation of midbrain DA neurons in rodents, results in reduced adult neurogenesis both in striatum and hippocampus^24,27^.

The dopamine transporter (DAT) constitutes one regulatory mechanism of extracellular DA and altered DAT functioning has been linked to several neuropsychiatric diseases with dysregulation of DA neuronal function^28^. Rats overexpressing the DAT (DAT-tg) display profound alterations within the DA system, i.e. increased striatal and hippocampal D1/D2 receptor expression as well as decreased striatal DA and two-fold increased hippocampus DA content^29^. In addition, DAT-tg rats exhibit increased hippocampal volumes suggesting also functional consequences within hippocampal circuitry. So far, neurobiological alterations were shown to translate into repetitive behaviour. We here studied whether these profound alterations in striatal and hippocampal DA homeostasis also affect hippocampal adult neurogenesis translating into learning and memory deficits.

## Results

**Figure 1:**
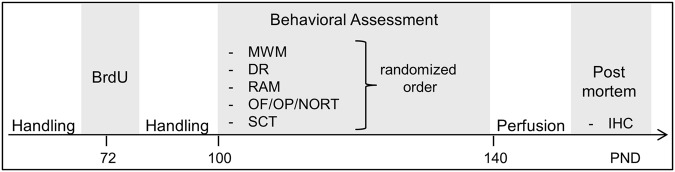
Experimental design. Labelling of newly generated cells for analysis of hippocampal neurogenesis was achieved by injecting proliferation marker BrdU into rats, three times with a six hours interval at a dose of 50 mg/kg. Following BrdU injections, animals performed a series of tasks investigating aspects of learning and memory and sensorimotor function. Behavioural experiments were performed in 3 batches of animals (A: wt n = 4, het n = 8; B: wt n = 2, het n = 4;C: wt n = 5, het n = 4). Test order Batch A: RAM, DR, SCT, OF/OP/NOR, MWM; Batch B: RAM, OF/OP/NOR, MWM, SCT, DR; Batch C: MWM, RAM, OF/OP/NOR, SCT, DR. BrdU = 5-Bromo-2′Deoxyuridine, MWM = Morris water maze, DR = Discrimination Learning, RAM = Radial arm maze, OF = open –field, OP = open platform, NOR = novel object recognition, SCT = sucrose consumption test, PND = postnatal day, IHC = immunohistochemistry.

Experiments were conducted on male hemizygous dopamine transporter overexpressing rats (DAT-tg) and their respective control littermates (wildtypes, wt) as outlined in Fig. 1.

### Morris water maze (MWM)

#### Acquisition training

**Figure 2:**
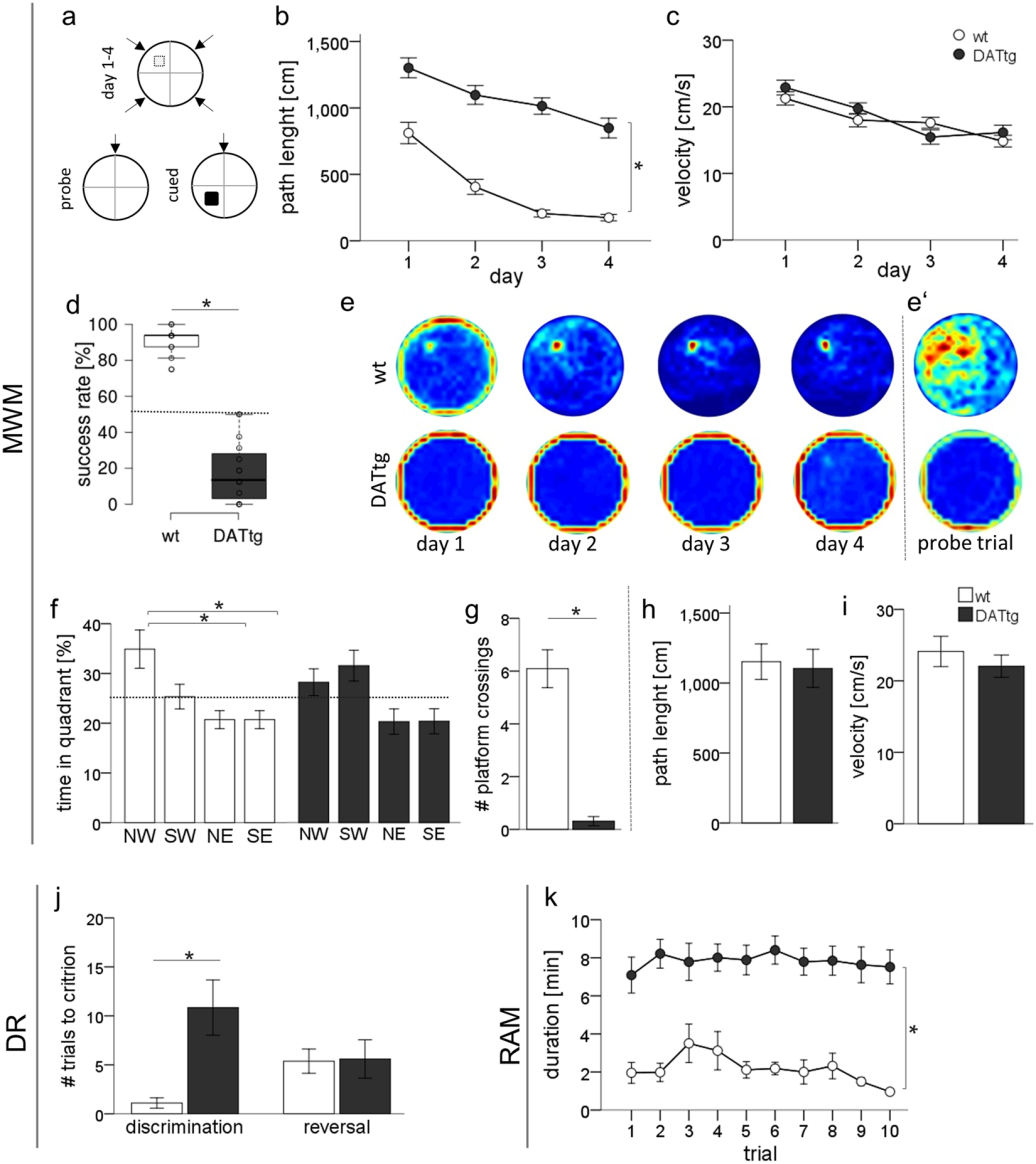
Behavioural assessment of learning and memory. (**d**) Schematics of MWM set up. In the Acquisition phase (day1–4) DAT-tg rats do not learn to locate the hidden platform (**a**), thereby showing longer path length compared to controls (**b**) but intact motor function (velocity (**c**)). (**e**) The probabilistic occupancy plots represent sum data over trials and rats within respective groups and illustrate the rapid development of a place-specific preference for the platform position for control animals but not DAT-tg animals. (**e**–**g**) In accordance to the impairment in learning during acquisition DAT-tg animals do not prefer the former goal quadrant after platform removal as found for controls. (**e**) During probe trial DAT-tg rats continuously show thigmotactic swimming around the wall of the pool. DAT-tg: n = 16; wt: n = 11 (**h**,**i**) Performance during cued platform trials indicates intact sensorimotor function when platform is visible. DAT-tg: n = 10; wt: n = 8 (**j**) During the discrimination learning paradigm DAT-tg rats exhibit impairments in initial acquisition. DAT-tg: n = 9; wt: n = 11 (**k**) During RAM DAT-tg rats exhibit significantly longer trial durations due to lack in exploratory behaviour. DAT-tg: n = 16; wt: n = 11 Dashed line (**a**,**f**) indicates chance level. Error bars indicate the standard error of the mean, significance level *p* < 0.05.

Control animals successfully learned to find the hidden platform during the acquisition period. In comparison DAT-tg animals showed a significantly lower rate of successful navigation to the platform (trial: *F*(6.8,171) = 3.516, *p* = 0.002; genotype: (*F*(1, 25) = 181.2, *p* < 0.001; trial × genotype interaction: *F*(6.8, 171) = 3.941, *p* = 0.001; repeated measures ANOVA) and none of the DAT-tg animals has been found to reach the platform above chance level (Fig. 2d). In accordance path length was significantly increased in DAT-tg animals compared to wt with significant differences on all days of the acquisition phase (day: *F*(2.4,59.8) = 47.61, *p* < 0.001; genotype: *F*(1, 25) = 78.673, *p* < 0.001; day × genotype interaction: *F*(2.4,59.8) = 3.655, *p* = 0.025; repeated measures ANOVA; Fig. 2b). Both, a significant trial × genotype interaction for successful navigation to the platform and a significant day × genotype interaction for path length, reflect a substantial learning defect i.e. shallow learning curve in DAT-tg rats. Latency to find the platform could not be analysed due to the few numbers of successful trials exhibit by DAT-tg animals. However DAT-tg animals performed equally in regard to swim speed (day: *F*(3,75) = 17.612, *p* < 0.001; genotype: *F*(1,25) = 0.571, *p* > 0.05; day × genotype, *F*(3,75) = 1.797, *p* > 0.05; repeated measures ANOVA; Fig. 2c).

#### Spatial search strategies

At each trial in the course of learning in the water maze, animals have a specific probability to choose an effective, more hippocampus-dependent spatial search strategy that depends on the already learned spatial knowledge over a less hippocampus dependent nonspatial strategy^30^. To assess such qualitative aspects of learning during acquisition training, swimming paths were categorized into different behavioural strategies, representing progression from thigmotaxis to direct swimming (namely: thigmotaxis, random search, scanning, chaining, directed search, focal search and direct swimming^31^). As illustrated through visual inspection of probability plots (Fig. 2e) control animals were found to proceed reliably from initial thigmotaxic behaviour towards using allocentric strategies, where distal cues provide geometric reference to the animal’s location while DAT-tg rats failed to do so. Using a repeated measures logistic regression model, we statistically assessed changes in the chance (odds) for using either a more hippocampus-dependent or less hippocampus-dependent strategy comparing DAT-tg to wt rats. We found a statistically significant effect on strategy choice for the use of more hippocampus versus less hippocampus dependent strategies on genotype (*Estimate*_*genotype*_ = −0.81, *SE* = 0.22, *z* = −3.71, *p* < 0.001). The estimated odds-ratio (OR) was OR = 0.44(*p* < 0.001). Thus, DAT overexpression in DAT-tg rats significantly reduced the chance of an animal to use a spatial, more hippocampus-dependent strategy. We than specifically tested the use of thigmotaxis versus all other more complex strategies and found a statistically significant effect (*Estimate*_*genotype*_ = 1.07, *SE* = 0.23, *z* = 4.54, OR = 2.9, *p* < 0.001). Thus, in DAT-tg rats the chance to use thigmotaxis as a strategy is almost 3fold higher compared to wt.

#### Probe trial performance

On the fifth day when the platform had been removed wt rats spent most of the time in the previous goal quadrant indicative of successful spatial learning (NW/NE *t*(10) = 3.452, *p* = 0.007; NW/SE *t*(10) = 2.303, *p* = 0.047; all other *p* > 0.05; Student’s t-test, Fig. 2f). In contrast, DAT-tg rats did not exhibit significant preferences for any of the pool quadrants (all *p* > 0.05; Student’s t-test, Fig. 2f) and showed significantly fewer crossings over the former platform position compared to controls (*U*(27) = −4.559, *p* < 0.001; Fig. 2g). Again DAT-tg animals differed by significantly exhibiting thigmotaxic swimming while controls exploited egocentric and allocentric search strategies (*U*(27) = −4.941, *p* < 0.001; Fig. 2e).

#### Cued platform trial

Performance in cued trial was not significant different in DAT-tg compared to wt rats. Independent of the genotype not all animals were found to successfully approach the platform within 60 s (*χ*^2^(1) = 1.8, *p* = 0.178). Further similar path length (*t*(16) = 0.250, *p* = 0.806; Fig. 2h,) and velocity (*t*(16) = 0.806, *p* = 0.435; Fig. 2i) support the notion that sensorimotor function cannot account for the spatial learning and retention deficits observed in the DAT-tg animals.

### Discrimination reversal (DR)

The DR paradigm initially requires learning to discriminate the favourable T-Maze arm providing the escape platform. 43.8% of DAT-tg rats dropped out at this stage (n = 3 drowning/not swimming, n = 4 did not reach criterion) while all control rats reached criterion of 5 consecutive correct choices within 25 trials. ANOVA for animals completing the task showed a significant difference between genotypes; that is DAT-tg animals needed significantly more trials for discrimination learning than wt rats (*U*(20) = −2.381, *p* = 0.022; Fig. 2j). Performance during the reversal stage, which reflects the ability to change behaviour in the face of altering contingencies did not significantly differ between genotypes (*U*(20) = −0.956, *p* = 0.356; Fig. 2j).

### Radial maze

DAT-tg exhibited lower explorative behaviour already during habituation trials, i.e. they did not explore all arms and therefore did not consume all baits. During test trials DAT-tg animals continually showed a significantly lower rate of successful completion of the task over all trials i.e. location and consumption of all baits within 10 min (*U*(27) = −3.461, *p* < 0.001). This was caused by a reduced exploration behaviour i.e. staying in one arm over the whole trial duration. In accordance trial duration was significantly increased in DAT-tg compared to wt rats, with no improvement over task progression (day x genotype: *F*(4, 92) = 1.22, *p* = 0.278; genotype: *F*(1, 23) = 46.799, *p* < 0.001; day: F(4, 92) = 1.232, *p* = 0.308; repeated measures ANOVA; (Fig. 2k). The observed highly reduced engagement of DAT-tg animals in task activity impeded further analysis of working memory and reference memory errors.

### Exploratory behaviour

**Figure 3:**
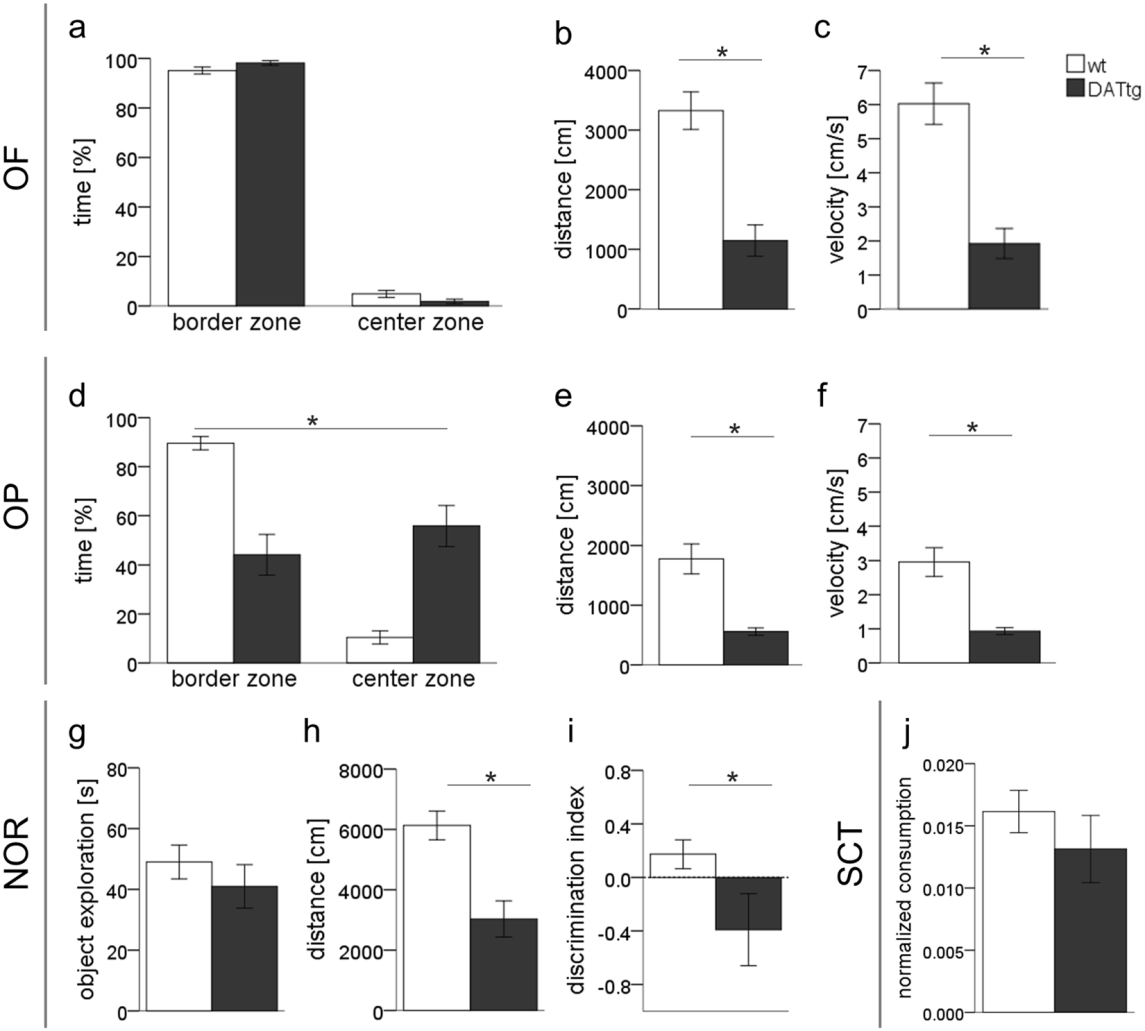
Exploratory behaviour and general locomotor activity. Results from the (**a**–**c**) Open-field (OF) (**d**–**f**) Open-platform (OP) (**g**–**i**) novel object recognition (NOR) and (**j**) Sucrose consumption test (SCT) are presented. (**i**) Discrimination index can vary between +1 and −1, where a positive score indicates more time spent with the novel object for controls, and a negative score for DAT-tg rats indicates more time spent with the familiar object. The dashed line indicates a null preference of novel-object investigation. (**a**–**f**,**j**) DAT-tg: n = 16; wt: n = 11 (**g**–**i**) DAT-tg: n = 7; wt: n = 11 Error bars indicate the standard error of the mean, significance level *p* < 0.05.

Analysis of general locomotor activity in the open field (OF) revealed a significant difference in genotypes for distance travelled (*t*(25) = 5.302, *p* < 0.001; Fig. 3b) and velocity (*t*(25) = 5.611, *p* < 0.001; Fig. 3c). As expected rats, independent of their genotype did spend a significantly greater amount of time in the wall zone of the arena. The time spend in centre zone did not differ between genotypes (*t*(25) = 1.860, *p* = 0.075; Fig. 3a). Analogous in the open platform (OP) test locomotor activity was reduced in DAT-tg rats; distance travelled (*t*(25) = 0.415, *p* = 0.001; Fig. 3e), velocity (t(25) = 5.534, *p* = 0.001; Fig. 3f). Further a significant difference was found for the time spend in border and centre zone, respective (*t*(25) = 4.399, *p* < 0.001; Fig. 3d).

During the familiarization phase of the novel object recognition (NOR) test individual DAT-tg animals were found to explore only one of the two objects while over the group this preference was not biased for object or object location. Accordingly overall distance travelled (*t*(17) = 2.832, *p* = 0.011; Fig. 3h) was significantly reduced in DAT-tg compared to wt rats however the total time spend with object exploration was not different (*t*(17) = 0.583, *p* = 0.568; Fig. 3g). During test-phase one-sample Wilcoxon rank test revealed that the average discrimination index (DI) was not significantly above or below chance level for neither wt nor DAT-tg rats (*p* > 0.05). However DAT-tg compared to control exhibit a reduced novel-object preference (*t*(17) = 2.192, *p* = 0.045; Fig. 3i).

No difference between genotypes was found in sucrose consumption (*t*(25) = 0.865, *p* = 0.396; Fig. 3j).

### Hippocampal neurogenesis

**Figure 4:**
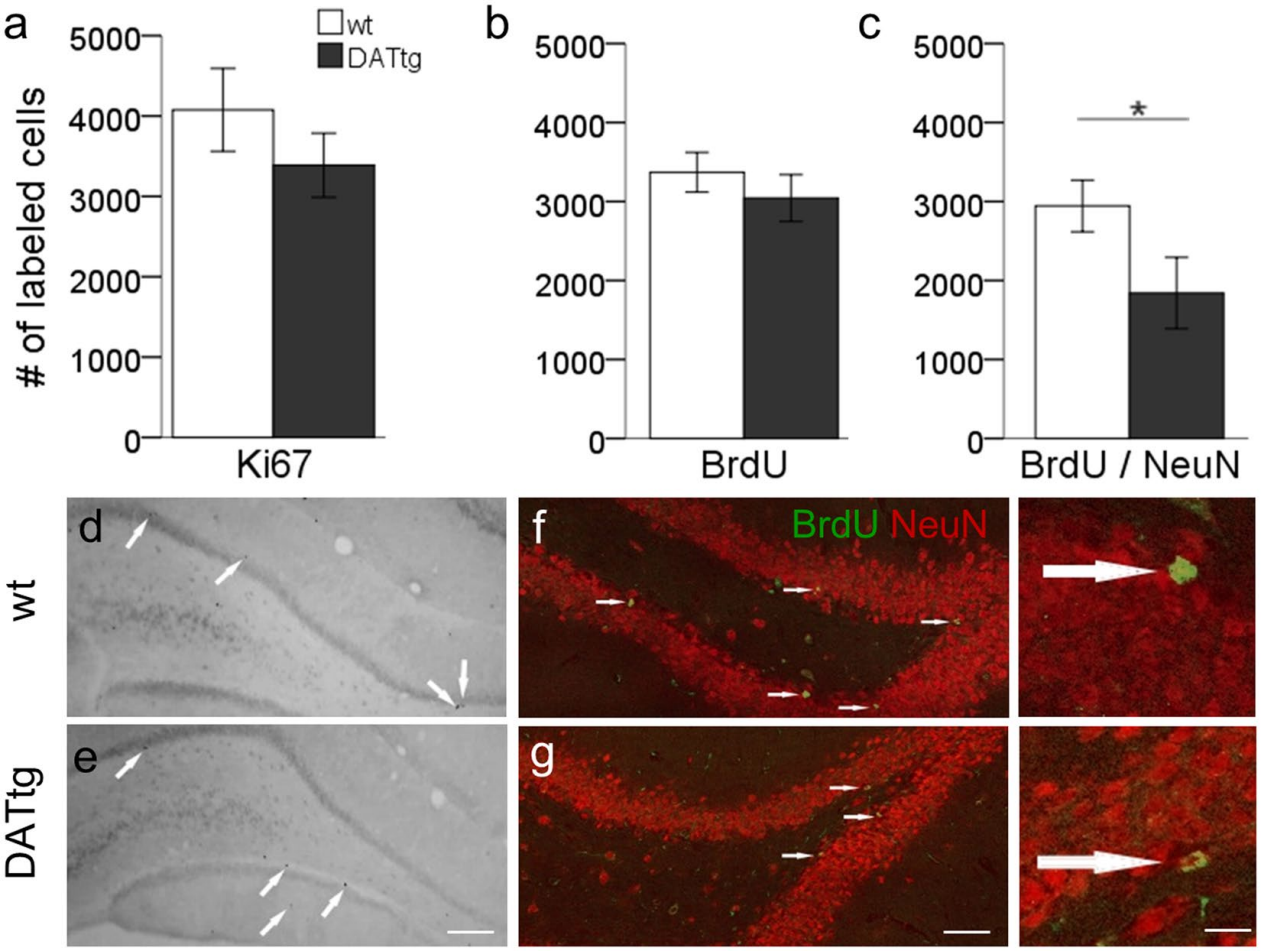
Histochemical analysis of hippocampal neurogenesis. Ten weeks after injection and behavioural assessment proliferation (Ki67) survival of newly generated cells (BrdU) and proportion of generated neurons (BrdU/NeuN) was quantified. The number of (**a**) Ki67-positive cells for DAT-tg: n = 9; wt: n = 7 and (**b**) BrdU-positive cells. DAT-tg: n = 13; wt: n = 7 did not differ between genotypes. However in DAT-tg rats lower numbers of BrdU/NeuN double-labelled cells could be detected. DAT-tg: n = 13; wt: n = 7. Error bars indicate the standard error of the mean, significance level p < 0.05. (**d**–**g**) Representative images for Ki67 DAB staining (**d**,**e**) and BrdU/NeuN immunofluorescent staining (**f**,**g**) are shown. Ki67 bright field, NeuN, red; BrdU, green; Scale bar: (**d**,**e**) 150 µm, (**f**,**g**) 100 µm, (insets) 15 µm.

Quantitative analysis of active proliferating progenitors in the SGZ of the dentate gyrus (DG) following our extensive behavioural experimental program was done using Ki-67, an endogenous marker for proliferating cells. There was no difference in the number of Ki-67+ cells between DAT-tg and wt rats (*t*(14) = 1, *p* = 0.332; students-t test; Fig. 4a). Similarly no significant difference was found in the number of bromodeoxyuridine (BrdU) labelled cells 10 weeks post injection (*t*(18) = −0.891, *p* = 0.385; students-t test; Fig. 4b). However co-labelling analysis with the mature neuronal marker NeuN (BrdU^+^/NeuN^+^) showed a significant reduction in the neuronal BrdU^+^ population in DAT-tg compared to wt rats (*t*(18) = 2.140, *p* = 0.046; students-t test; Fig. 4b), indicative of reduced incorporation of newly generated neurons into hippocampal circuitry in DAT-tg rats.

## Discussion

DAT-tg rats displayed immense deficits in acquiring information as well as a reduced integration of newly generated neurons into hippocampal circuitry. Balanced DA levels are crucial for cognitive performance and both too little and too much DA impairs performance e.g. for reward-based learning^32^.

Previously, dopamine deficient mice have been shown to not engage in behaviours in which food is used as reinforcement^33^. Likewise, DAT-tg rats were unable to perform in the radial arm maze. DAT provides a rapid and efficient mechanism for reuptake of synaptic DA. DAT-overactivity consequently causes exceptionally fast DA reuptake and therefore rapid clearing of DA from synapses, modelling synaptic DA deficiency. Changes in DA levels have repeatedly been reported to immediately affect willingness to engage in work, supporting the idea that fast DA fluctuations influence motivational aspects of decision-making^34^. Correspondingly, as seen in the OF as well as the NORT, novelty was not sufficient to provide motivation to move in the DAT-tg rats. While sucrose consumption data indicates a similar hedonic impact, altered DA and its motivational function i.e. the willingness to engage in work to receive the reward^35^ may underlie the reduced performance of DAT-tg animals in the appetitive RAM.

In contrast in the MWM and the discrimination reversal water comprises a strongly aversive component providing the means to motivate movement (swimming) as a necessity to approach the hidden platform. Nevertheless DAT-tg rats were severely compromised also in performance in these tasks. In the MWM, thigmotaxis, which is swimming along the walls of the pool, was the most prominent behaviour seen in DAT-tg rats. Initial thigmotaxis is commonly observed in rats but is usually rapidly replaced by efficient cognitive strategies that depend on the association between environmental cues and the spatial location of the platform. Also in the present study, control rats showed an immediate shift to approach the platform limiting thigmotactic behaviour to the first trials and exhibiting a steep learning curve. DAT-tg animals however showed continuous thigmotaxis over all trials and thus were severely impaired in locating the hidden platform. There are several explanations for excessive thigmotaxis found in the literature: motor impairments, lack of orientation, increased anxiety or an inability to deploy spatial search strategies. With respect to motor impairments analysis of open field behaviour showed no apparent defects in coordination. Additionally, when placed in water DAT-tg rats were capable of swimming with normal swim speed. DAT-tg rats further displayed normal sensorimotor function indicated by visual performance using optomotor tracking (Supplement) and adequate performance using the visible platform as an intra-maze cue thus demonstrating a general awareness of surroundings and orientation. Additionally, DAT-tg rats in a previous study scored normal in an anxiety paradigm^29^.

Evidence from our search strategy analysis rather suggests that DAT-tg rats were unable to deploy spatial search strategies. Mura and Feldon^36^, who performed 6-OHDA lesions of the nigrostriatal system, abolishing DA signalling, were the first to suggest that excessive thigmotaxis results from an impairment to choose the correct strategy to solve the task. More recently a similar conclusion was derived following lesion experiments to the dorsomedial striatum^37^. Consequently, an aberrant striatal DA state may at least partially explain the observed learning deficits.

An alternative explanation is given by the observation of DA changes in the DAT-tg rat translating into a stress and amphetamine induced repetitive behaviour phenotype^29^. Pathological repetitive behaviour can be exacerbated by specific environmental and psychological triggers, including sensory stimulation frustration, anxiety or stress^38^. As the DAT-tg model has been discussed to represent a model of repetitive phenotypes and the aversive environment in the MWM represents a well-known stress inducing factor, the observed thigmotactic swimming may alternatively represent a repetitive behaviour. Within the present behavioural scheme we did not observe excessive grooming or other types of repetitive behaviour however as it was not systematically assessed its occurrence cannot be fully excluded. Such hypothesis thus remains to be further investigated.

Behavioural analysis additionally suggests an inability to acquire spatial reference memory (probe trial defects) thus evidence for a hippocampal dependent learning deficit. In line with the literature DA plays an important role in the spatial components of learning^13,39–41^. Such hippocampus-dependent learning is sustained by continuous cell rearrangement via adult neurogenesis and the several steps of adult generation of neurons, i.e. proliferation, differentiation and functional integration, which have been shown to be modulated by DA signalling. The observed behavioural defect suggests that changes in dopamine homeostasis in the DAT-tg model may affect hippocampal adult neurogenesis contributing to the spatial learning deficits. We do not see a reduction in proliferation in our model, though we do find a reduced proportion of newly born functional integrated (BrdU+/NeuN+) neurons, indicative of reduced incorporation of newly generated neurons into hippocampal circuitry in DAT-tg rats. An alternative explanation may be a general reduction of mature hippocampal neuronal circuitry in DAT-tg rats as a result of continuous DAT overexpression, which yet seems unlikely as prior analysis has not found differences in NeuN expressing cells in several brain regions including the hippocampus between DAT-tg and control rats^29^. Further experiments however need to evaluate causality between reduced numbers of newly integrated neurons and impaired learning considering that DAT-tg animals displayed lower physical activity in the testing situation, which can reduce integration of newly generated neurons^42^.

## Conclusion

Given the important role of DA signalling for the ability to execute proper learning functions, altered DA homeostasis, hippocampal structures and disinhibition of the striatum may in combination underlie impaired performance in the DAT-tg rats. DA homeostasis clearly constitutes a critical component of the cellular network sub serving information processing per se but may be similarly essential for the proper development of such a network during embryogenesis, postnatal or even adult stages.

## Materials and Methods

### Animals

All animal experiments were carried out in accordance to the European Communities Council Directive of 22th September 2010 (2010/63/EU) under protocols approved by the animal ethics committees of the Technische Universität Dresden and the Landesdirektion Sachsen. Animals were generated in our lab as described elsewhere^29^. Briefly a construct containing the NSE promoter, murine DAT coding sequence, and bovine growth hormone polyadenylation sequence was used for microinjection into the pronucleus of zygotes from Sprague-Dawley (SD) Hanover rats (Janvier labs). Transgenics are maintained on SD-background in a continuous hemizygous x wildtype offspring breeding scheme (>20 generations). Genotypes were verified using PCR. Animals were housed in mixed genotype groups of two–four in a 12-h light dark cycle (light on at 06:00 am) with food and water ad libitum. All efforts were made to reduce animal suffering and number of animals used.

### Experimental design

Animals were BrdU injected to quantify adult neurogenesis (Fig. 1). Animals were habituated to the experimenter during 5-min handling sessions over 3 consecutive days prior to injections. Rats were injected with BrdU (50 mg/kg) every 6 hours over a period of eighteen hours (three injections total). The majority of adult born neurons die before they mature, the surviving neurons are functionally integrated into existing neural circuits within one month^43–45^. The rate of survival of newborn neurons is regulated by experiences, including hippocampus-dependent learning^46,47^. As we aimed to assess learning in relation to baseline neurogenesis rather than performance-depended hippocampal neurogenesis we chose to start behavioural experiments when labelled neurons are functionally integrated. Thus, testing took place between 4–10 weeks post injection, which also represents a time window with a stable number of BrdU-labelled cells^45^. Animals where scarified immediately following the last behavioural experiment and brains processed for post mortem analysis.

### Behavioural testing

Testing took place in three parallel batches with distinct test orders. Animals never performed more than one test a day and between each behavioural test animals were allowed to rest for 3–5 days to reduce stress and support recovery e.g. following weight loss from food deprivation. All behavioural testing took place between 10:00 and 16:00 h. Experimenters were blind to the genotype of the rats during all experimental sessions. If not otherwise stated all behavioural data were collected using EthoVisionXT video tracking equipment and software (Noldus Information Technology) at a rate of 5 frames per second. Learning was assessed using the Morris water maze, radial arm maze and discrimination reversal. Open field analysis, novel object recognition and sucrose consumption tests were included to assess factors that may interfere with learning performance such as locomotion, anxiety, novelty response and taste perception.

#### The Morris water maze

Hidden platform test was used to investigate spatial learning and memory. A pool (diameter 1.6 m) was positioned in a room with distal cues visible to the swimming animal. Water in the pool was maintained at 24 °C (±1 °C), filled to a depth of 33 cm and made opaque by non-toxic white paint. A small platform (14 × 14 cm) was hidden 1 cm beneath the water surface. For analysis, the pool was divided into four quadrants (northwest (NW), southwest (SW), northeast (NE) and southeast (SE)) with the platform being located in NW over all acquisition trials (Fig. 2a). Rats accomplished four trials per day with a 60 s trial limit, in which they had to find the platform followed by a 5 s resting period (on platform) before being removed (inter-trial interval (ITI) of 15 min). Acquisition trials lasted for 4 days, i.e. leading to 16 trials in total. Each day rats were released at four different starting positions randomized over days. If a rat failed to find the platform within the time limit on the first trial on the first day, it was led to the platform. On the fifth day, a 60 s probe trial was performed from a novel start position with the platform removed. Latency (time required to find the hidden platform), mean velocity (swimming speed) and path length (length of path swum by the animal in one trial) were recorded. Acquisition trials were further analysed to identify differential search strategies according to previously described methods^31^. Seven main search strategies were identified ranging from thigmotactic behaviour (rats swimming predominantly close to the wall) to non-spatial strategies (i.e. scanning) to proper spatial strategies (i.e. swimming directly to the platform). During the probe trial, time spent in the former target quadrant and former platform crossings were recorded.

As a control condition, frequently used in the MWM, cued platform trials were performed with a set of animal’s naïve to the spatial version of the MWM. Cued trials require identical basic prerequisites such as vision, motor performance (swimming, climbing onto the platform) and motivation to escape as spatial trials. Each animal performed one trial with a 60 s trial limit where the platform was placed 1 cm above the water within the SE quadrant and marked with a balloon hanging 10 cm above the platform.

#### Discrimination Reversal

was assessed in a T-maze filled with water maintained at 25 °C (±1 °C), with a hidden platform (15.5 × 15.5 cm) in one of the arms. On the first day (*position discrimination)* rats were trained to acquire left-right position discrimination with the platform consistently positioned in one of the arms. Rats were allowed to choose between arms. Once entered an arm, a door was lowered. If the correct arm was chosen, the rat was allowed to remain on the platform for 5 s, if the wrong one was chosen, the rat was confined to the arm for 5 s. Training continued with a 10 s inter-trial interval until a criterion of five consecutive correct trials was reached within a maximum of 25 trials. On the next day *(reversal)*, rats were first retrained until criterion on the position discrimination of the first day was reached, and then trained until reaching the criterion on the reversal of this discrimination, i.e. with the platform located in the opposite arm. The number of trials to reach the criterion was recorded for both sessions.

#### Radial arm maze

Starting two days before RAM the animals were restricted to approximately 20 g of rat chow per day. The rat’s weight was monitored daily to ensure that their health was maintained. An endpoint of 20% weight loss was established; which was not reached thus no animals had to be removed from the study. The RAM apparatus was elevated 65 cm above the floor, consisted of a central platform (47 cm diameter) with eight arms (40 × 15 cm) radiating from it. The apparatus was positioned in a room with distal cues on the walls visible to the animal. One day prior to testing, the baits used (Choco Krispies, KELLOG) were presented to the animals in their home cage. On day 1 to day 3, animals were allowed to explore the maze freely for 10 min or until consuming the baits hidden in the wells at the end of each arm. On day 4 to day 8, three of the arms were baited, randomly assigned but consistent for each animal over all trials. Again, animals were removed after 10 min or after finding all hidden baits. Training took place twice a day. The time spend in the maze, entered arms and the order of entry were recorded, including reference memory errors, i.e. entry into a non-baited arm, and working memory error, i.e. entry into a previously visited arm.

#### Open-field

An adjusted version was conducted to measure general locomotor activity and anxiety. The arena consisted of a square open-field box (70 × 70 × 40 cm) constructed of grey PVC plastic and evenly illuminated. On the first day the box was used as a platform (standing on its walls) placed elevated 1 m above the floor. The platform was covered with tissue to prevent slipping. Animals were released in the middle of the platform to explore freely for 10 mins. On the second day, the same box was used now placed on a table with the surrounding walls up. The animals were released in the middle and again, allowed to explore for 10 min. The arena was cleaned with 70% EtOH between each rat. A camera was positioned over the arena and behaviour was recorded. For both sessions, open-field (OF) and open-platform (OP), a square of 20 × 20 cm in the middle of the arena was designated as the centre and time spent in border and centre zone was analysed. Additionally, distance travelled and velocity were recorded.

#### The novel object recognition

test was used to evaluate the rat’s ability to recognize a novel object in the environment without positive or negative reinforcers thereby assessing the natural preference for novelty displayed by the animals^48^. The task procedure consisted of three phases: habituation, familiarization, and test phase. Open-field analysis were conducted the day before NOR, and thereby considered as habituation to the test environment^49^. During familiarization, two objects (*A* + *A′*) different in colour and size were placed in the OF arena on opposite corners with a distance of 20 cm from the walls. Animals were released in the middle of the box facing the opposite wall and could familiarize with the objects for 5 min. After a 24 h retention interval, the animals returned to the arena, were now one object was familiar ($A$) and the other object was replaced with a novel object, again different in form and colour ($B$). During the test phase animals were allowed to explore for 5 min. All stimuli consisted of objects made of glass, porcelain, or glazed ceramic and were cleaned with 70% EtOH between each rat. A video camera was positioned over the arena and familiarization and test phases were videotaped for analysis. Time spent exploring each object was measured by two blinded experimenters (within-session inter-rater reliability was moderate *r* = 0.752, *p* < 0.001, range: 0.703–0.840). Exploration was defined as sniffing or touching the object in a radius of 0 to 4 cm with its nose. Climbing and sitting on the object and touching it with the body was not considered exploration. Animals lacking exploration activity i.e. did not spend a minimum of 7 s exploring either object during familiarization phase (9 animals all DAT-tg), were excluded from analysis^50^. The main dependent measure the Discrimination Index was calculated from the exploration time $T$ as $\mathrm{DI}=({T}_{B}-{T}_{A})/({T}_{B}+{T}_{A})$ based on the 5 min of the test phase, averaged over two independent assessors. Further distance travelled and velocity was analysed.

#### Sucrose consumption test (SCT)

assesses an animals’ response to a stimulus that should be perceived as rewarding. Rats were habituated to single cages and bottles containing sweetened condensed milk (Milchmädchencreme, Nestle; 1:3 mix with water) for 30 min each, 48 h and 24 h before testing, respectively. Following food restriction (15 g food/rat/24 h) rats were exposed to the sweetened bottles in single cages for 10 min. Bottles were weighed before and after the test session and the amount of liquid consumed was normalized to each animal’s mean body weight, measured over the three consecutive days.

### Post mortem neurobiological assessment

#### Tissue collection

Rats were transcardially perfused, brains removed and post-fixed overnight in 4% paraformaldehyde. 40 µm coronal sections were cut on a freezing microtome and a series of every sixth section was used for respective analysis.

#### Immunohistochemistry

Staining were carried out using standard protocols on free-floating sections. Sections for BrdU staining were pre-treatment with 2 N HCl for 30 min at 37 °C. Multiple washes in phosphate-buffered saline were performed between all further steps. After blocking with 10% donkey serum containing 0.2% Triton X-100, sections were incubated overnight with primary antibodies (for BrdU: rat anti-BrdU, AbD Serotec OBT0030, Cambridge, United Kingdom, 1:500; for NeuN: mouse anti-NeuN, Millipore, MAB377, 1:500; for Ki-67+: NCL-Ki67p, Novocastra Laboratories, Newcastle upon Tyne, UK, 1:500) in blocking solution containing 3% donkey serum and 0.2% Triton X-100. Ki67 and BrdU samples were detected with anti-rat or anti-rabbit-biotin coupled secondary antibodies (both 1:500; Dianova) together with the horseradish peroxidase-coupled ABC Elite system (Vector Laboratories, USA) and visualized with 3,3′-diaminobenzidine (Sigma) and 0.04% NiCl as the chromogen before counting under a light microscope. BrdU/NeuN double-labelled samples were detected with fluorescent secondary antibodies (donkey anti-rat Alexa Fluor 488, donkey anti-mouse Cy3 and donkey anti-rabbit Alexa Fluor 647; Jackson ImmunoResearch, UK), the nuclei counterstained with 4′,6-diamidino-2-phenylindole, and then visualized for counting using an ApoTome fluorescence microscope (Zeiss, Germany) with Optical Sectioning mode (Structured Illumination Microscopy). Sampling of labelled cells was done exhaustively throughout the GCL in its rostro-caudal extension. A simplified version of the optical fractionator principle was used where labelled cells were categorized according to their localization in the dentate gyrus and counted except for cells in the uppermost focal plane to avoid oversampling at the cutting surfaces^51^. The resulting number was than multiplied by 6 (because every sixth section had been used) to give an estimate of the total number of positive cells. All counts were carried out with the experimenter blind to the experimental group.

### Statistical analysis

Group differences were tested using two-tailed t-tests or nonparametric Mann-Whitney-U test when applicable. Repeated measures ANOVA models were used for variables taken repetitively on the same animal, such as trial or day, as within-subject factors. Main effects were Bonferroni adjusted, if applicable the Greenhouse–Geisser adjustment was used to correct for violations of sphericity, post-hoc tests applied Bonferroni correction (SPSS; IBM Corp. Released 2013, IBM SPSS Statistics for Windows, and Version 22.0. Armonk, NY: IBM Corp). The probability level of *p* < 0.05 was considered as statistically significant. Data are presented as mean ± SEM. For statistical analyses of the effect of genotype on search strategy, we used binomial (logit) mixed-effects models (glmer, package: lme4; R 3.4.3 (https://www.r-project.org/)) predicting strategy probabilities (0 vs.1) for genotypes (0.5 = het vs. −0.5 = wt). A maximum random effects structure was used^52^. From the model odds ratios were calculated to compare the chance of using divergent strategies between genotypes.

## Electronic supplementary material

Supplementary Information

## Data Availability

The datasets generated during and/or analysed during the current study are available from the corresponding author on reasonable request.
