# Supplementary material for: Learning deficits in rats overexpressing the dopamine transporter
Source: Sci Rep. 2018 Sep 21;8:14173. doi: 10.1038/s41598-018-32608-7 (PMC6154965; doi:10.1038/s41598-018-32608-7)
Supplement: Supplementary file 1 — Supplementary Information [file 41598_2018_32608_MOESM1_ESM.doc]

# Supplement

# Learning deficits in rats overexpressing the dopamine transporter

^1^Nadine Bernhardt, ^1^Maike Kristin Lieser, ^1,2^Elizabeth Barroeta Hlusicka, ^1,2^Bettina Habelt

^1,2^Franziska Wieske , ^1,2^Henriette Edemann-Callesen, ^3^Alexander Garthe, ^1,2^Christine Winter*

### Affiliation

^1^Department of Psychiatry and Psychotherapy, University Hospital Carl Gustav Carus, Technische Universität Dresden, Germany

^2^ Department of Psychiatry and Psychotherapy, Charité Universitätsmedizin Berlin, Germany.

^3^ German Center for Neurodegenerative Diseases (DZNE) Dresden, Dresden, Germany.

* Corresponding author E-mail:

Prof. Dr. Christine Winter, Department of Psychiatry and Psychotherapy, Charité University Medicine Berlin, Charitéplatz 1, 10117 Berlin, Germany. E-mail: christine.winter@charite.de; Tel: ++4930450517310; Fax: ++4930450525919

Optomotor Tracking

Visual ability was assessed by means of optomotor tracking using a stripe pattern that slowly rotates around the animals´ head. A functioning visual system is thereby proven through minimal reflexive turning of the animals´ head in direction of the stripes´ rotation, the so called optomotor reflex. This head turning reflex is predominantly mediated by the optokinetic and vestibulo-ocular reflexes stabilizing the image of the environment on the retina (Cahill and Nathans, 2008).

**Method** One animal at a time was placed on a platform within the measuring chamber in the middle of a slowly rotating drum (OptoDrum, Striatech, Tübingen, Germany). Through an integrated camera above the platform the tracking system detected the animal and started a digital presentation of rotating black-and-white vertical stripes. In random intervals of a few seconds rotation switched direction. Head movements were automatically detected through the tracking system and double-checked by two independent raters. A visual system test was assessed as successful if in more than 50 % of sessions a head turning was detected.

**Results** Optomotor tracking was performend with three rats each, wildtypes and DAT-tg. While one control animal failed the test all DAT-tg rats passed the test indicating a functional visual system.

**Reference** Cahill H, Nathans J (2008) The Optokinetic Reflex as a Tool for Quantitative Analyses of Nervous System Function in Mice: Application to Genetic and Drug-Induced Variation. PLoS ONE 3(4): e2055. doi:10.1371/journal.pone.0002055
